# New Aspects of an Old Drug – Diclofenac Targets MYC and Glucose Metabolism in Tumor Cells

**DOI:** 10.1371/journal.pone.0066987

**Published:** 2013-07-09

**Authors:** Eva Gottfried, Sven A. Lang, Kathrin Renner, Anja Bosserhoff, Wolfram Gronwald, Michael Rehli, Sabine Einhell, Isabel Gedig, Katrin Singer, Anton Seilbeck, Andreas Mackensen, Oliver Grauer, Peter Hau, Katja Dettmer, Reinhard Andreesen, Peter J. Oefner, Marina Kreutz

**Affiliations:** 1 Department of Hematology and Oncology, University of Regensburg, Regensburg, Germany; 2 Regensburg Centre for Interventional Immunology (RCI), University of Regensburg, Regensburg, Germany; 3 Department of Surgery, University of Regensburg, Regensburg, Germany; 4 Institute of Pathology, University of Regensburg, Regensburg, Germany; 5 Institute of Functional Genomics, University of Regensburg, Regensburg, Germany; 6 Department of Internal Medicine 5, Hematology/Oncology, University of Erlangen, Erlangen, Germany; 7 Department of Neurology, University of Muenster, Muenster, Germany; 8 Department of Neurology, University of Regensburg, Regensburg, Germany; 9 Wilhelm Sander NeuroOncology Unit, University of Regensburg, Regensburg, Germany; Instituto Nacional de Cardiologia, Mexico

## Abstract

Non-steroidal anti-inflammatory drugs such as diclofenac exhibit potent anticancer effects. Up to now these effects were mainly attributed to its classical role as COX-inhibitor. Here we show novel COX-independent effects of diclofenac. Diclofenac significantly diminished MYC expression and modulated glucose metabolism resulting in impaired melanoma, leukemia, and carcinoma cell line proliferation *in vitro* and reduced melanoma growth *in vivo.* In contrast, the non-selective COX inhibitor aspirin and the COX-2 specific inhibitor NS-398 had no effect on MYC expression and glucose metabolism. Diclofenac significantly decreased glucose transporter 1 (*GLUT1*), lactate dehydrogenase A (*LDHA*), and monocarboxylate transporter 1 (*MCT1*) gene expression in line with a decrease in glucose uptake and lactate secretion. A significant intracellular accumulation of lactate by diclofenac preceded the observed effect on gene expression, suggesting a direct inhibitory effect of diclofenac on lactate efflux. While intracellular lactate accumulation impairs cellular proliferation and gene expression, it does not inhibit MYC expression as evidenced by the lack of MYC regulation by the MCT inhibitor α-cyano-4-hydroxycinnamic acid. Finally, in a cell line with a tetracycline-regulated *c-MYC* gene, diclofenac decreased proliferation both in the presence and absence of c-MYC. Thus, diclofenac targets tumor cell proliferation via two mechanisms, that is inhibition of MYC and lactate transport. Based on these results, diclofenac holds potential as a clinically applicable MYC and glycolysis inhibitor supporting established tumor therapies.

## Introduction

The transcription factor MYC plays a key role in the regulation of cell growth, differentiation and apoptosis [1]. Normal cells are characterized by low steady state levels of MYC expression. This tight control is lost in many human malignancies leading to high constitutive expression of MYC. The inactivation of MYC can revert the neoplastic phenotype in tumor model [2]. Therefore, MYC represents an attractive target for cancer therapy in humans [3]; [4], but currently no MYC inhibitor other than dexamethasone is clinically applicable.

Overexpression of MYC leads to the upregulation of glycolytic enzymes such as glucose transporter-1 (GLUT1) and lactate dehydrogenase-A (LDHA) [5]; [6]. High rates of glucose uptake and glycolysis are characteristic for human cancers, a feature already described by Otto Warburg almost a century ago [7]–[9]. High lactate concentrations in the tumor correlate with malignancy [10] and genetic downregulation of LDHA results in reduced tumor growth *in vivo* [11]; [12]. Furthermore, pharmacological targeting of glucose metabolism by 2-deoxyglucose (2-DG) and 3-bromopyruvate (3-BrPA), both inhibitors of glycolysis, as well as dichloroacetate (DCA), which targets the mitochondrial pyruvate dehydrogenase kinase (PDK), has been shown to reduce tumor growth in animal models [13]–[16]. In addition, silencing of the lactate transporters (monocarboxylate transporter, MCT) by shRNA *in vitro and in vivo* results in a reduction of cell viability and tumor growth [17]; [18]. Lactate transport can also be blocked by pharmacological means as non-steroidal anti-inflammatory drugs (NSAIDs) have been shown to reduce the transport of lactate in a human trophoblast cell line and chinese hamster ovary (CHO) cells [19]; [20]. However, this effect of NSAIDs has never been addressed with regard to inhibition of tumor growth, although several epidemiological studies report that the use of NSAIDs is linked to a lower risk of inflammation-associated tumors like colon, oesophagus and breast cancer [21]; [22]. The relationship between chronic inflammation and cancer has already been described by Virchow in 1863 and is still accepted as an important component of tumor development [23]. Anti-tumor effects of NSAIDs have been attributed mainly to the inhibition of cyclooxygenase (COX1/2) and their anti-inflammatory effects, albeit COX-independent inhibition of tumor cell proliferation and induction of apoptosis have been also reported [24]; [25]. In addition, it has been known for years, that NSAIDs affect mitochondrial activity and function and this aspect has recently been linked to its anti-proliferative effect on tumor cells [26]; [27].

Here, we show a novel COX-independent effect of the NSAID diclofenac on human and murine tumor cells via reduction of MYC, glucose uptake and lactate secretion. Since tumor cell proliferation was diminished *in vitro* and growth of subcutaneous tumors was impaired *in vivo*, diclofenac might be used as a novel anti-cancer drug supporting established tumor therapies.

## Materials and Methods

### Chemicals and Drugs

All drugs were purchased from Sigma (Taufkirchen, Germany) and dissolved in water, unless otherwise indicated. The sodium salt of diclofenac, acetylsalicylic acid (aspirin, ASA) (both from Fagron, Barsbüttel, Germany), NS-398, gemcitabine (Hospira, Munich, Germany), and alpha-cyano-4-hydroxycinnamic acid (CHCA) (Sigma) were dissolved in medium.

### Cells and Cell Culture

The human melanoma cell line MelIm [28] was obtained from Judith Johnson, Institute for Immunology, Munich, Germany, in 1993 and has been tested in the last 3 months for melanocyte markers and melanoma markers by RT-PCR. B16 subclone of B16F10 mouse melanoma [29]. The human histiocytic leukemia cell line U937 was purchased from DSMZ (German Collection of Microorganisms and Cell Cultures, Braunschweig, Germany). B16 and U937 were identified by DSMZ in May 2011. The prostate carcinoma line PC3 and the T-cell leukemia cell line Jurkat were purchased from ATCC. P493-6, a B cell line carrying a conditional, tetracyclin-regulated MYC gene, was provided by G. W. Bornkamm Munich, Germany [30]. All cell lines were cultured in RPMI 1640, 10% fetal calf serum (both from PAN Biotech, Germany), 2 mM glutamine, 50 U/mL penicillin/50 µg/mL streptomycin (all from Gibco) at 5% CO_2_ and 37°C. For suppression of MYC, P493-6 cells were treated with 1 µg/mL tetracycline for 24 h before diclofenac treatment.

Monocytes were obtained by leukapheresis of healthy donors, followed by density gradient centrifugation over Ficoll/Hypaque and separation by countercurrent centrifugation (J6M-E centrifuge; Beckmann, Munich, Germany). Monocytes were cultured at a concentration of 1×10^6^ cells/ml for 48 h in RPMI 1640, 10% fetal calf serum (both from PAN Biotech, Germany), 2 mM glutamine, 50 U/mL penicillin/50 µg/mL streptomycin (all from Gibco) and 100 ng/ml LPS at 5% CO_2_ and 37°C in teflon bags with or without the addition of diclofenac.

### Determination of Cell Proliferation

3×10^4^ cells/0.2 mL medium were incubated for 22 h with diclofenac, ASA or gemcitabine in 96-well plates. [^3^H]-thymidine incorporation was determined 24 h after the addition of 0.5 µCi/0.2 mL [^3^H]-thymidine (Amersham Pharmacia, Piscataway, NJ).

### Determination of Apoptosis

For analysis of apoptosis, diclofenac treated cells were stained with Annexin-V-FITC and 7-aminoactinomycin D (7-AAD) (both from BD Biosciences, San Jose, CA) according to the manufacturer's instructions. Flow cytometric analyses were performed on a FACSCalibur (BD Biosciences) using BD CellQuestPro for data acquisition and analysis.

### Subcutaneous Tumor Mouse Model

Animal experiments were approved by the Institutional Animal Care and Use Committee of the University of Regensburg and regional authorities. In brief, 1×10^5^ cells of B16 were subcutaneously injected into the right flank of C57/BL6 mice (Charles River, Sulzfeld, Germany). Mice were randomized and assigned to treatment groups (n = 7/group). Once tumor volumes reached 50–80 mm^3^ (day 14), mice received diclofenac (15 mg/kg) or saline via intraperitoneal (i.p.) injection every other day until termination of the experiment on day 23. 15 mg/kg diclofenac corresponds approximately to 0.5 mM used for in vitro assays. Tumor diameters were measured with calipers and tumor volumes were calculated (width^2^ × length ×0.5).

### Determination of Lactate in Tumor Cell Supernatants

Cells were seeded at a concentration of 2×10^5^ cells/2 mL medium with or without diclofenac, ASA or gemcitabine. After 48 h, lactate levels in cell culture supernatants were determined by means of an ADVIA1650 analyzer (Bayer, Tarrrytown, NY) using reagents from Roche (Mannheim, Germany) at the Department of Clinical Chemistry, University Clinic, Regensburg, Germany.

### Western Blotting

2,5×10^6^ cells/4 mL medium were cultured in 6-well plates over night. Whole cell lysates were prepared with RIPA-buffer and samples (50 µg) were subjected to western blotting on a denaturating 10% SDS-PAGE. Membranes were sequentially probed with antibodies against MYC (#9402, Cell Signaling Technologies, Beverly, MA), STAT3 (Cell Signaling Technologies, Beverly, MA), HIF-1a (sc-10790, Santa Cruz Biotechnologies, Santa Cruz, CA), HIF-2a (NB100-132, Novus Biologicals, Littleton, CO) or β-Actin (Santa Cruz Biotechnologies, Santa Cruz, CA) in dry milk (5%) and detection was performed by chemoluminescence (ECL, Amersham Bioscience, Piscataway, NJ). Densitometric analyses were performed by means of the ChemiDoc MP Imaging System and the Image Lab^TM^ software (Bio-Rad Laboratories, Hercules, CA).

### Promoter Assay

The region upstream the transcription start site of human *MYC* (2632bp, chr8:128746062-128748693) was amplified from genomic DNA and cloned into the Luciferase Reporter Vector pGL4 (Promega). MelIm were cotransfected in 6-well-plates with the luciferase construct or the empty pGL4 vector (Promega) and cotransfected with an internal control vector (phRL-TK, Promega) using Lipofectamine™ 2000 (Invitrogen). Diclofenac was added after 5 h at different concentrations. 24 h after transfection, luciferase activity was determined in cell lysates using the Dual-Luciferase-Reporter Assay System (Promega) according to the manufacturer's instructions. The activity was normalized by the ratio of Firefly luciferase activity to Renilla luciferase activity (internal control) and compared to pGL4 empty vector.

### RNA Isolation and Quantification of mRNA Expression

2.5×10^6^ cells/4 mL medium were incubated for 24 h in 6-well plates. Total RNA was isolated using the RNeasy Mini Kit (Qiagen, Germany). After reverse transcription using M-MLV reverse transcriptase (Promega, Germany), products were analyzed on a Mastercyler Ep Realplex (Eppendorf, Germany) using the QuantiFast SYBR Green PCR Kit (Qiagen, Germany). Expression data were normalized to the housekeeper 18S rRNA. Primers used were 5′-3′:

*18S rRNA* sense: ACCGATTGGATGGTTTAGTGAG; *18S rRNA* antisense: CCTACGGAAACCTTGTTACGAC; *GLUT1* sense: AACTCTTCAGCCAGGGTCCAC; *GLUT1* antisense: CACAGTGAAGATGATGAAGACGTAGGG; *LDHA* sense: GGTTGGTGCTGTTGGCATGG; *LDHA* antisense: TGCCCCAGCCGTGATAATGA.

### NMR Spectroscopy

For determination of glucose levels in cell culture supernatants, cells were cultured at 2×10^6^ cells/4 mL medium with or without diclofenac for 48 h. 1D and 2D NMR spectra of the filtered supernatants were measured on a 600 MHz Bruker Avance III spectrometer (Bruker BioSpin GmbH, Rheinstetten, Germany) as described previously [31].

### Gas Chromatography – Mass Spectrometry (GC-MS)

U937 cell pellets, washed 3 times with PBS, were extracted using 2 mL 80% methanol. MelIm cells were harvested by directed scraping with 80% methanol as described before [32]. During extraction, samples were spiked with 10 μL of a surrogate solution containing [U-^13^C]lactate (Euriso-top, Saint-Aubin Cedex, France) and [^2^H_4_]diclofenac (CDN Isotopes Inc., Quebec, Canada) at a concentration of 1 mM each.

Dried sample extracts were analyzed by GC-MS in full scan mode [33]. Quantification of lactate was performed using calibration curves and diclofenac by stable isotope dilution using the mass trace of m/z 214 for diclofenac and m/z of 218 for [^2^H_4_]diclofenac.

### Respirometry

Activity of the respiratory system was analyzed in a two-channel titration injection respirometer (Oxygraph-2k, Oroboros, Innsbruck, Austria) at 37°C. Cells were resuspended in fresh culture medium and added to the chamber. After a stabilization phase of 15 to 20 min, ROUTINE respiration of intact cells was measured, complex V was inhibited by oligomycin (2 µg/mL), and subsequently the capacity of the electron transfer system (ETS) was determined after uncoupling with FCCP (2.5 µM). Residual oxygen consumption was determined after addition of rotenone (complex I inhibitor, 0.1 µM) and antimycin A (complex III inhibitor 2.5 µM) and subtracted from all respiratory parameters.

### Statistical Analysis

All results represent mean +/− standard deviation (SD) of at least three independent experiments. Statistical analysis was performed with unpaired, two-tailed Student's t-test, ***p<0.001; **p<0.01; *p<0.05. For western blots, one representative experiment is shown.

## Results

### Diclofenac inhibits melanoma cell proliferation *in vitro*

**Figure 1:**
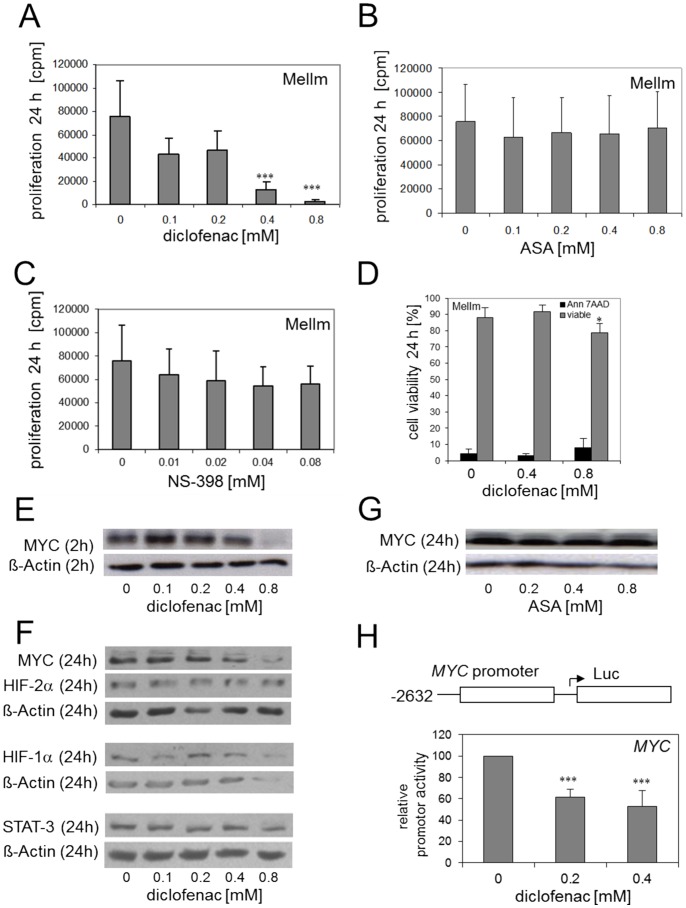
*In vitro* effects of diclofenac on proliferation and MYC expression in the human melanoma cell line MelIm. The human melanoma cell line MelIm was incubated with different concentrations of diclofenac (A), aspirin (ASA, B), and NS-398 (C), respectively, and proliferation was determined after 24 h. Results represent the mean +/− standard deviation of 12 (diclofenac) and 3 (ASA, NS-398) independent experiments, respectively. (D) MelIm were incubated for 24 h with or without diclofenac. Apoptotic cells were stained with Annexin-V-FITC/ 7-AAD and analyzed by flow cytometry. Results represent the mean +/− standard deviation of 3 independent experiments. (E-G) MYC, STAT3, HIF1a and HIF2a protein expression were determined in cell lysates of MelIm incubated for 2 or 24 h with or without diclofenac (E,F) or ASA (G). The effect of diclofenac on MYC promoter activity was determined by transient transfection of a 2632-bp MYC promoter fragment (H). MelIm were transfected in 6-well-plates and diclofenac was added after 5 h. Luciferase activity was determined 24 h after transfection. Results represent the mean +/− standard deviation of 3 independent experiments.

The addition of diclofenac, which is a member of the arylacetic acid group of NSAIDs, at clinically relevant concentrations (see http://www.drugs.com/pro/diclofenac.html) led to significant effects on several tumor cell lines starting at concentrations as low as 0.1 mM. The proliferation of the human melanoma cell line MelIm was inhibited significantly (p<0.001) at 0.4 mM diclofenac (Fig. 1A) and reduction in proliferation was comparable to that of the standard chemotherapeutic drug gemcitabine (Fig. S1A In contrast, aspirin (ASA), a typical NSAID exerted no impact on proliferation indicating a COX-independent effect of diclofenac (Fig. 1B). Other NSAIDs like the COX-2 specific inhibitor NS-398 (Fig. 1C), as well as 4-ASA and 5-ASA (data not shown) also exerted no significant impact on tumor cell proliferation. To clarify whether the effect of diclofenac on proliferation is based on the induction of cell death, we analyzed viable and apoptotic cells after incubation with diclofenac in MelIm. Only 0.8 mM diclofenac exerted a slight but significant effect on the number of dead cells after 24 h (Fig. 1D).

### Diclofenac inhibits MYC expression in melanoma cells

It is well known, that tumor cell proliferation is associated with an upregulation of oncogenes like MYC and that the inactivation of MYC can revert the neoplastic phenotype and induce apoptosis [2]. Therefore, we analyzed the expression of MYC protein under the administration of diclofenac by western blot analysis. After 2 h and 24 h, we observed a clear reduction in MYC protein level in MelIm cells (Fig. 1E/F and Fig. S2A) which coincided with the inhibition of proliferation. In contrast, ASA did not affect MYC expression (Fig. 1G). The protein expression of other transcription factors, namely STAT3, a known critical regulator of melanoma development [34], and HIF-1α and HIF-2α, respectively, were not influenced by diclofenac (Fig. 1F). As Zhu et al. had described modulating, albeit stimulatory effects of indomethacin not only on MYC protein but also on MYC gene expression [35], we performed a MYC promoter assay. We cloned a region of about 2.6 kb upstream the transcription start site of human MYC into the Luciferase Reporter Vector pGL4 and transfected the plasmid into MelIm. As shown in Figure 1H, diclofenac suppressed MYC promoter activity significantly.

### Diclofenac inhibits MYC expression and proliferation in leukemia and carcinoma cells

**Figure 2:**
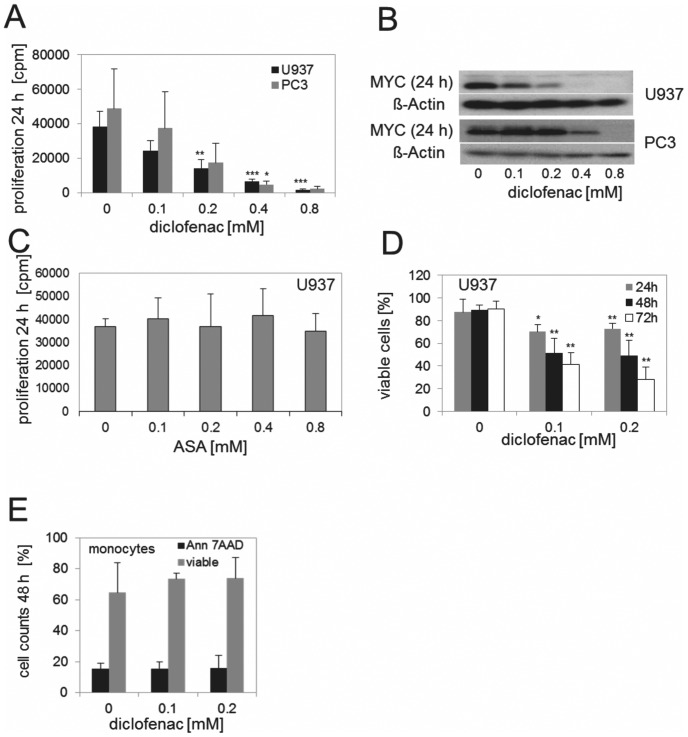
Effects of diclofenac on different tumor cell lines. (A) U937 and PC3 were incubated with increasing concentrations of diclofenac and proliferation was determined after 24 h. Results represent the mean +/− standard deviation of at least 3 independent experiments (PC3: 0.8 n = 2). (B) MYC protein expression was analyzed by western blotting in cell lysates of U937 and PC3 after 24 h incubation with diclofenac. (C) U937 was incubated with increasing concentrations of aspirin (ASA) and proliferation was determined after 24 h. Results represent the mean +/− standard deviation of 3 independent experiments. (D) U937 were incubated for 24 h up to 72 h with or without diclofenac and stained with Annexin-V-FITC/7-AAD. Results represent the mean +/− standard deviation of percentage of viable cells (Annexin-V-FITC/7-AAD negative cells) of 3 independent experiments. (E) Freshly isolated human monocytes were cultured for 48 h in the presence or absence of diclofenac, stained with Annexin-V-FITC/7-AAD and analysed by flow cytometry. Results represent the mean +/− standard deviation of percentage of viable cells (Annexin-V-FITC/7-AAD negative cells) and dead cells (Annexin-V-FITC/7-AAD positive cells) of 3 independent experiments.

To confirm that the effect of diclofenac on proliferation and MYC expression was not restricted to melanoma cells, we analysed its impact on the human histiocytic lymphoma cell line U937. Again, we found a significant inhibition of proliferation and MYC expression by diclofenac (Fig. 2A/ 2B and Fig. S2A) whereas aspirin (ASA) did not show an effect (Fig. 2C). Similar results were obtained with other tumor cell lines such as the prostate carcinoma cell line PC3 (Fig. 2A/B) and the T-cell leukemia cell line Jurkat (data not shown). In the myeloid leukemia cell line U937, diclofenac concentrations up to 0.4 mM only slightly reduced the number of viable cells after 24 h (about 80% viable cells left). This effect was more pronounced after 72 h reducing the number of viable cells to 45% and 31% for 0.1 mM and 0.2 mM, respectively (Fig. 2D). In contrast, the viability of non-malignant blood monocytes was not impaired even after prolonged incubation at these concentrations (Figure 2E).

### Diclofenac inhibits tumor cell proliferation *in vivo*

**Figure 3:**
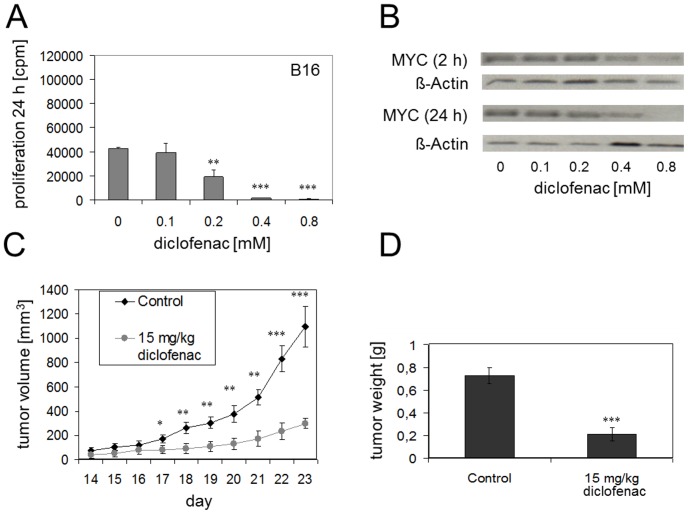
*In vitro* and *in vivo* effects of diclofenac on proliferation of B16 murine melanoma cells. (A) Proliferation of B16 cells was determined in the presence or absence of diclofenac after 24 h incubation. Results represent the mean +/− standard deviation of 4 independent experiments. (B) MYC expression was determined in B16 after incubation with diclofenac for 2 h and 24 h by western blotting. (C/D) For the analysis of in vivo effects of diclofenac on tumor growth, 1×10^5^ B16 cells were injected subcutaneously into C57/BL6 mice. At a tumor volume of 50–80 mm^3^ (day 14), mice received diclofenac (15 mg/kg, n = 7) or saline (control, n = 7) via intraperitoneal injection every other day. The tumor weight was determined on day 23 after termination of the experiment (D). Statistical analysis was performed with unpaired, two-tailed Student's t-test, ***p<0.001; **p<0.01; *p<0.05.

To demonstrate an *in vivo* effect of diclofenac, we switched to the mouse melanoma cell line B16. Diclofenac reduced proliferation significantly at concentrations of 0.2 mM and higher (Fig. 3A). Diclofenac also decreased MYC expression in a time- and dose-dependent fashion. Densitometric analyzes revealed that MYC protein was reduced starting at 0.2 mM diclofenac after 2 h incubation. This effect was more pronounced after 24 h, however a strong reduction was only detected at 0.4 mM and higher (Fig. 3B and Fig. S2B). Next we analyzed the effect of diclofenac on B16 tumor growth *in vivo* employing a syngeneic subcutaneous mouse model. On day 14, after tumors had reached a volume of 50–80 mm^3^, diclofenac (15 mg/kg) or saline were injected intraperitoneally every other day. Tumor growth was significantly impaired already after 3 days of diclofenac treatment (Fig. 3C), and so was the final tumor weight (Fig. 3D).

### Diclofenac inhibits glucose uptake and lactate secretion in tumor cells

**Figure 4:**
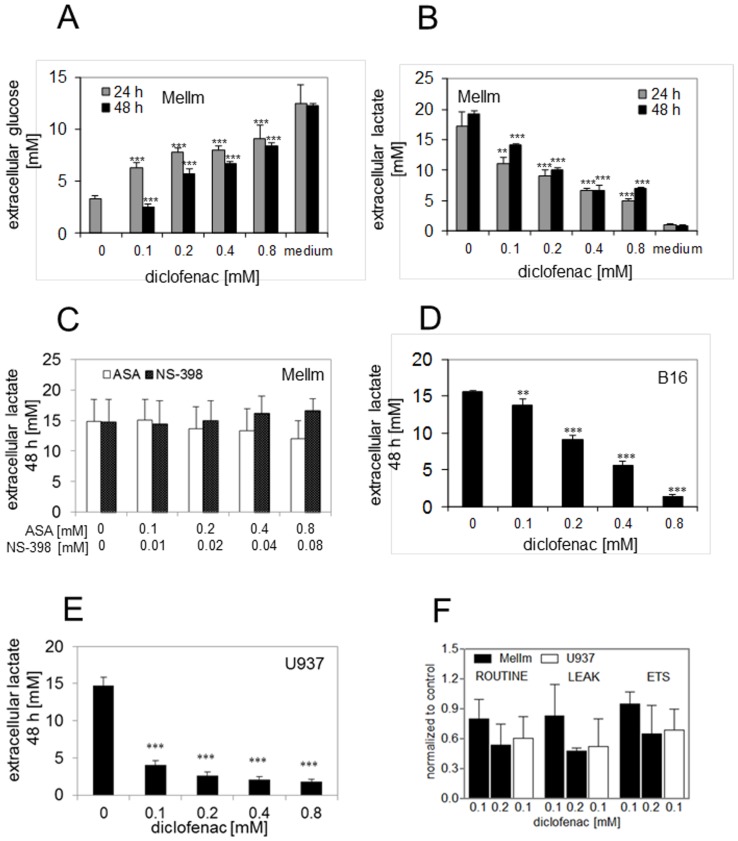
Diclofenac decreases glucose consumption and modulates lactate accumulation. (A) Glucose levels were determined in MelIm supernatants after 24 h and 48 h of incubation with diclofenac. “Medium” represents the glucose concentration in the culture medium without cells. Lactate was determined in cell culture supernatants of MelIm after 24 h and 48 h with or without diclofenac,(B) ASA or NS-398 (C). Lactate was determined in cell culture supernatants of B16 after 48 h (D). Lactate levels were determined in supernatants of U937 (E) after incubation with increasing concentrations of diclofenac for 48 h. Results represent the mean +/− standard deviation of 3 independent samples. Diclofenac reduced mitochondrial activity in both cell lines after 24 h (F). A reduction of about 40% was observed in MelIm at a concentration of 0.2 mM and in U937 at a concentration of 0.1 mM. Basal mitochondrial activity (ROUTINE), oligomycin inhibited respiration and capacity of electron transfer system (complex I to IV) were diminished by diclofenac. Results represent the mean+/−SD of 3 and 5 independent experiments, respectively, for MelIm and U937.

Given the known promoting effect of MYC on glycolysis [5], we analyzed glucose metabolism in different cell lines. First, we analyzed glucose consumption in the presence of diclofenac. After 24 h and 48 h we found a significant inhibition of glucose uptake in MelIm already at 0.1 mM diclofenac (Fig. 4A). In most tumor cells, glucose is predominantly metabolized to lactate [8]. Lactate has to be secreted to avoid intracellular accumulation and acidification, which blocks ATP generation via glycolysis. We determined lactate in the supernatant of diclofenac treated MelIm, B16 and U937. Compared to control, significantly lower extracellular lactate levels were observed for MelIm at diclofenac concentrations as low as 0.1 mM at 24 h and 48 h, respectively (Fig. 4B). In contrast, neither aspirin (ASA) nor NS-398 (Fig. 4C) affected extracellular lactate levels. Accordingly, B16 and U937 also yielded lower levels of extracellular lactate (Fig. 4D and 4E). The effect of diclofenac was not due to a reduction in cell number, as the classical chemotherapeutic drug gemcitabine inhibited proliferation but not lactate secretion (Fig. S1A/B).

Diclofenac diminished glucose uptake and thereby substrate delivery for mitochondrial respiration. Moreover, it was shown that diclofenac inhibits pyruvate uptake into mitochondria. Therefore, we measured mitochondrial respiration by high-resolution respirometry at diclofenac concentrations that sufficed to diminish glucose uptake but did not exhibit effects on proliferation proliferation Immediately after addition of diclofenac we detected an increase in basal respiration (ROUTINE) in both cell lines (MelIm 54%, U937 39%). In U937 the increase in respiration was the result of decoupling (detected by an equally increase in oligomycin inhibited respiration) and was reversible. In MelIm elevated respiratory activity was a combination of increased activity coupled to ATP production and decoupling. After 24 h of diclofenac treatment ROUTINE respiration and oligomycin inhibited respiration were significantly suppressed in U937 at 0.1 mM diclofenac and in MelIm at 0.2 mM diclofenac (p<0.05, Fig. 4F). The capacity of the electron transfer system showed the same tendency, but was statistically not significant.

### Diclofenac blocks lactate transport and leads to intracellular lactate accumulation

**Figure 5:**
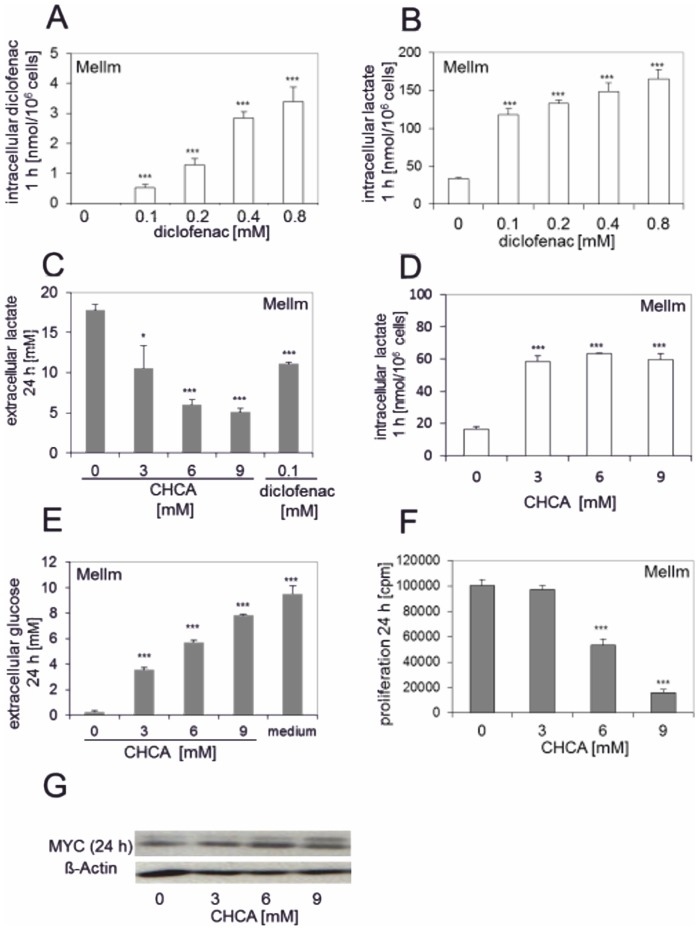
Blocking lactate transport by CHCA inhibits proliferation and lactate secretion in MelIm, but has no effect on MYC expression. The intracellular concentration of diclofenac in the cell lysates was determined after 1 h incubation (A). Intracellular lactate levels were determined in cell lysates of MelIm after 1 h incubation with or without diclofenac (B). Results represent the mean +/− standard deviation of 3 independent samples. Lactate was determined in cell culture supernatants of MelIm treated for 48 h with CHCA or diclofenac (C). Results represent the mean +/− standard deviation of 4 independent samples. Intracellular lactate levels were determined in cell lysates of MelIm after 1-h incubation with CHCA (D). Glucose levels were determined in MelIm supernatants after 24 h incubation with CHCA (E). Proliferation of MelIm was determined after 24 h in the presence or absence of CHCA (F). Results represent the mean +/− standard deviation of 4 independent samples. MYC protein expression was analyzed in cell lysates of MelIm after 24 h incubation with CHCA by western blotting (G). One representative blot is shown.

Next, we analyzed the cause of reduced extracellular lactate levels upon treatment with diclofenac. Lactate is transported out of the cell by monocarboxylate transporters (MCTs) that rely on a concentration gradient of lactate and protons between the extracellular and the intracellular compartment [36]. NSAIDs with monocarboxylic acid structures such as diclofenac have been reported to inhibit MCTs [19]; [37]; [38]. Quantitative RT-PCR analysis revealed a constitutive expression of MCT1, glucose transporter-1 (GLUT1) and lactate dehydrogenase A (LDHA) in MelIm. The expression was upregulated after 24 h, which was significantly prevented by diclofenac treatment (Fig. S3A-C). We hypothesized, that diclofenac blocked efflux of lactate. Indeed, gas chromatography-mass spectrometry analysis revealed that starting from 0.1 mM, diclofenac is significantly taken up by MelIm and U937 after 1 h (Fig. 5A and S4A), which was paralleled by a marked intracellular accumulation of lactate (Fig. 5B and S4B).

### MCT inhibition blocks lactate transport but has no effect on MYC expression

The small-molecule competitive inhibitor of MCT, α-cyano-4-hydroxycinnamic acid (CHCA), showed effects comparable to diclofenac. As expected, the inhibitor blocked lactate transport as shown by lowered extracellular levels of lactate upon incubation of MelIm with CHCA (Fig. 5C) and led to an intracellular accumulation of lactate (Fig. 5D). Concomitantly, glucose uptake was inhibited (Fig. 5E). In addition, comparable to diclofenac, administration of CHCA at 6 mM and higher caused a strong inhibition of tumor cell proliferation (Fig. 5F). To test whether the inhibition of MCTs and the resulting accumulation of lactate were responsible for the regulation of MYC, we analysed MYC expression after incubation of MelIm with CHCA. In contrast to diclofenac, CHCA did not inhibit MYC expression (Fig 5G).

### Diclofenac blocks tumor cell proliferation via MYC-dependent and -independent mechanisms

**Figure 6:**
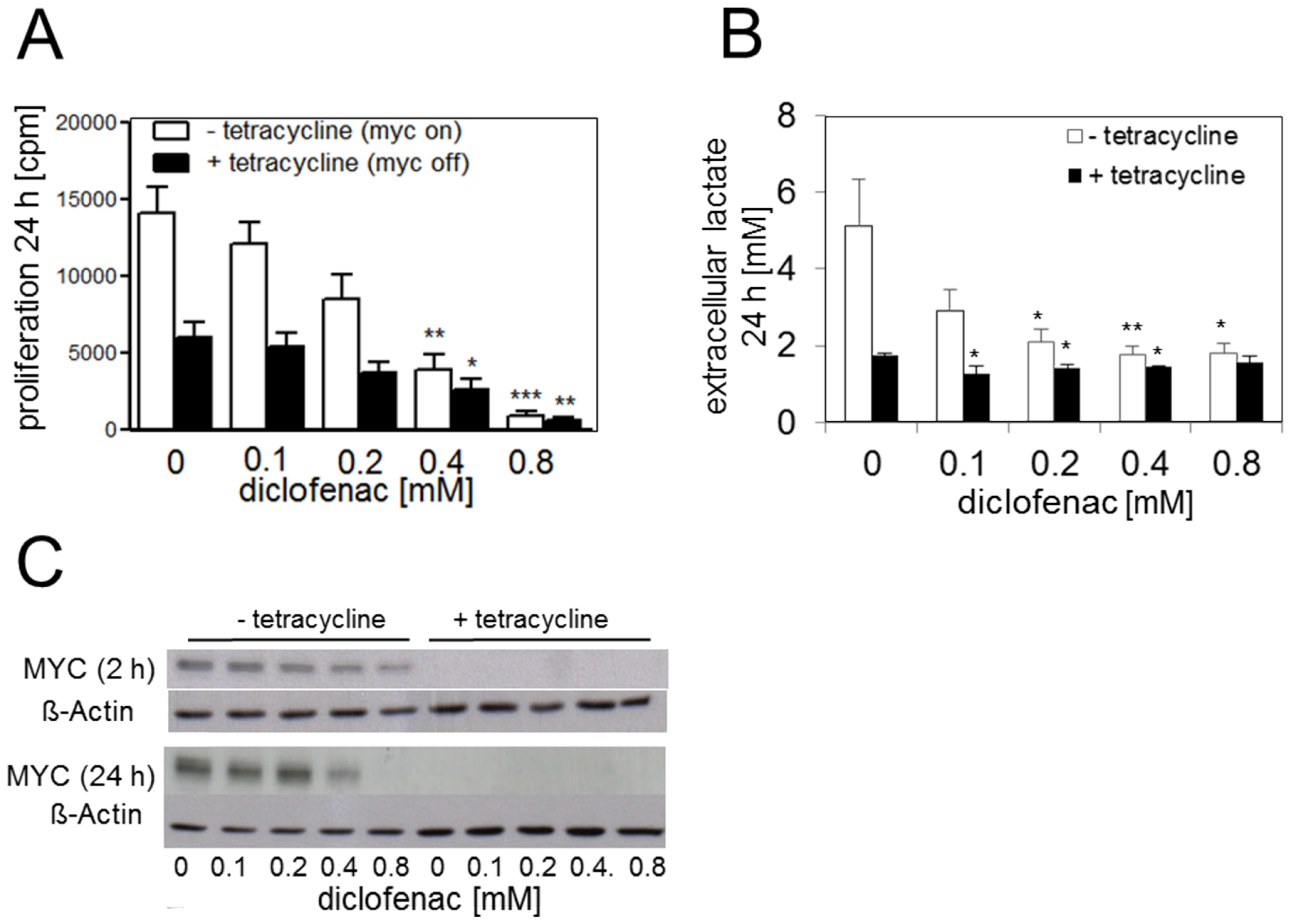
Contribution of MYC to suppression of proliferation by diclofenac. P493-6 cells were incubated first for 24 h in the presence of tetracycline to suppress MYC expression. Cells were then treated with diclofenac for 24 h. Proliferation (A) was determined by ^3^[H]-thymidine incorporation, while lactate (B) was determined in cell culture supernatants. Results represent the mean +/− standard deviation of 3 independent experiments. MYC protein expression was analyzed in cell lysates of P493-6 with or without tetracycline treatment for 24 h (C). Cells were then treated with diclofenac for an additional 2 h and 24 h, respectively, and MYC expression was determined in cell lysates by western blotting. One representative western blot is shown.

Our data indicate two independent effects of diclofenac on tumor cells that result in the inhibition of proliferation. To distinguish the relevance of MYC suppression and block of lactate transport on proliferation, we used human P493-6 B-cells, which are derived from human peripheral blood B cells immortalized by an Epstein–Barr viral (EBV) genome that is complemented with an EBV nuclear antigen-estrogen receptor (EBNA2-ER) fusion protein and a tetracycline-repressible MYC transgene [30]. In the absence of tetracycline and estradiol, ectopic MYC is induced at high levels, whereas with tetracycline only very low levels of MYC are expressed [39]. Concordingly, in the presence of tetracycline proliferation was about 2.5-fold lower than in its absence (Fig. 6A). Upon addition of increasing concentrations of diclofenac, proliferation decreased increasingly, albeit much more significantly in MYC overexpressing cells. In the absence of detectable MYC expression, lactate secretion was about 4-fold lower than in MYC overexpressing cells (Fig. 6B). Increasing concentrations of diclofenac inhibited significantly lactate secretion in MYC overexpressing cells, with extracellular levels of lactate approaching those observed in cells treated with tetracycline. As observed for MelIm, B16, and U937 cells, diclofenac reduced MYC protein levels in MYC overexpressing P493-6 cells after 2 h and 24 h (Fig. 6C). Further investigations will clarify whether this effect is based on modulation of protein stability or transcriptional regulation. These experiments confirm the ability of diclofenac to inhibit lactate secretion and MYC expression at concentrations of 0.1 mM and about 0.4 mM, respectively, with both effects contributing to reduced proliferation.

## Discussion

A hallmark of many cancer cells is an increased reliance on glycolytic metabolism and the production of large amounts of lactate regardless of the availability of oxygen. This so-called “Warburg effect” can be the result of biochemical and genetic alterations [40] such as HIF1 stabilization [41], loss of p53 [42] or a target gene of p53 [43], mutation of KRAS/BRAF [44] or overexpression of MYC [5]. Drugs targeting key control points of glycolysis are subject of intense research as promising anticancer agents. However, no drugs other than DCA and the synthetic glucocorticoids dexamethasone and prednisolone are available as glycolytic inhibitors for clinical use. Glucocorticoids are also potent MYC inhibitors, but a plethora of deleterious side effects impede their prolonged use. NSAIDs represent the most commonly used class of medication worldwide. In the light of our findings, diclofenac may represent an attractive novel inhibitor of glycolysis and MYC, which could easily be integrated in clinical trials with likely important implications in cancer therapy. However, NSAID use can be associated with adverse side effects, e.g. liver and kidney injury as well as gastrointestinal bleeding. Although the incidence is low, it will be important to determine the risk of diclofenac treatment in relation to its possible benefit [45]–[47].

It is well-known that NSAIDs affect tumor cell proliferation in COX-dependent and independent ways. However, little is known about their effect on glucose metabolism. We found a significant decrease in glucose consumption and lactate secretion in different tumor cell lines upon treatment with diclofenac. However, these effects seemed not to be related to the classical COX inhibition, as ASA had no impact on glucose metabolism and proliferation. Others reported a reduction in lactate secretion in MCF-7 breast cancer cells by ASA, albeit at a ten-fold higher concentration [48]. The anti-proliferative effect, however, was not responsible for the decreased glucose consumption and lactate secretion, as the classical chemotherapeutic drug gemcitabine strongly decreased proliferation without any effect on lactate secretion.

Besides the *in vitro* effects on melanoma and histiocytic lymphoma cell lines, we also observed a significant reduction in tumor growth in a syngeneic melanoma mouse model. Effects of diclofenac on tumor growth had been described before in a murine fibrosarcoma model, two xenograft models with human neuroblastoma cells and lung carcinoma cells, and in a rat model of early colon carcinogenesis [49]–[52]. However, the authors attributed the effect of diclofenac primarily to the regulation of lipid metabolism and COX inhibition. In the light of our results, diclofenac does not only target lipid metabolism but also glucose metabolism in tumor cells leading to reduced tumor growth as demonstrated recently in a mouse glioma model [53]. In this model, diclofenac had a significant effect not only on tumor cells, but also on tumor stromal cells, e.g. myeloid and lymphoid cells. This is in line with reports by Mayorek et al. who studied the effect of diclofenac in a murine model of pancreatic cancer. Here, the effect on tumor growth was linked to downregulation of VEGF and angiogenesis in the tumor [54]. As lactate is known to stimulate VEGF production in macrophages, a reduction of intratumoral lactate levels by diclofenac could contribute to the diminished VEGF production and angiogenesis in this model [54]; [55]. Furthermore, tumor-derived lactate is not only a modulator of stromal cells in the tumor environment, but it can also serve as a carbon source for fibroblasts and aerobic tumor cells [56]; [57]. Therefore, intratumoral lactate levels influence tumor growth via several pathways [58].

We identified two potential mechanisms underlying the suppression of proliferation and glucose metabolism by diclofenac. First, diclofenac inhibited lactate efflux and, consequently, caused an intracellular accumulation of lactate. Lactate is transported via proton-coupled monocarboxylate transporters (MCTs), that form heterodimeric complexes with the glycoprotein CD147 [36]; [59]. Our data suggest, that diclofenac may target this transport system. This is in line with reports from Emoto and Vellonen on NSAID-mediated inhibition of lactate transport in a trophoblast cell line and corneal epithelial cell lines [19]; [38]. Accordingly, application of the competitive MCT inhibitor CHCA could reproduce the effect of diclofenac, inhibiting both lactate secretion and proliferation. It is known, that CHCA not only targets MCT in the plasma membrane but also in the mitochondrial membrane with possible implications on mitochondrial functions [60]. In line with these findings, we also detected a transient increased respiration mainly due to uncoupling after short-term incubation with diclofenac followed by a decreased respiration after 24 h. Aspirin/ASA and other NSAIDs are known to uncouple mitochondrial energy metabolism [26]. However, as diclofenac and ASA both target mitochondria in a similar fashion but only diclofenac affected MYC expression, we assume that the regulation of oxidative metabolism had no impact on MYC expression in our model system. However, the reduction in mitochondrial activity might contribute to the anti-proliferative effect of diclofenac on tumor cells and support the induction of apoptosis as has been shown for T-cell lymphoma cells [27]; [61].

Interestingly, Pouysségur and coworkers recently showed, that combined silencing of *MTC1* and *MCT4* significantly reduced glycolytic flux and tumor growth *in vivo* [18]. These results clearly show that lactate efflux, glycolysis and tumor growth are closely related. Similar results were described by Mathupala *et al.*, who showed that downregulation of *MCTs* by small hairpin RNA inhibited glycolysis and induced cell death in a glioma cell line [17]. In addition, silencing of CD147, an accessory subunit of MCT1/4, also inhibited malignant melanoma growth [62]. Besides the rapid block of lactate transport, we found a lack of upregulation of *MCT1* mRNA that might have contributed to the described effect. But changes in gene transcription often do not correlate with changes in protein levels or enzyme activity and we cannot exclude that despite a significant downregulation of *MCT1* mRNA levels, protein levels are not altered. Furthermore, expression of *GLUT1* and *LDHA* was significantly decreased by diclofenac in a concentration-dependent manner indicating that diclofenac not only targeted lactate transport but also exerted a more global effect on glycolysis. This effect was already detectable at very low concentrations of about 0.1 mM diclofenac. However, in MelIm MYC expression was significantly decreased only at higher concentrations of about 0.8 mM diclofenac. Therefore, additional factors might be involved in the transcriptional regulation of *LDHA* and *GLUT1* (summarized in Table 1). High concentrations of diclofenac strongly suppressed MYC protein expression and promoter activity. This effect was not due to COX inhibition, as aspirin did not suppress MYC expression. Furthermore, the inhibition of lactate transport seems not to be involved in MYC regulation because CHCA did not change MYC expression. In addition, intracellular lactate accumulation was comparable for 0.2 mM and 0.8 mM diclofenac but MYC regulation was significantly different. Downregulation of MYC by diclofenac correlated with tumor cell proliferation. However, induction of cell death was only detected at 0.8 mM diclofenac, a level that completely suppressed MYC. At this high concentration, diclofenac also completely suppressed MYC in the MYC overexpressing cell line P493-6 indicating not only a transcriptional effect but also an impact on protein stability. As inhibition of proliferation was found in MYC overexpressing and non-expressing P493-6, we concluded that diclofenac targeted proliferation via both MYC-dependent and independent mechanisms. MYC overexpressing P493-6 showed higher lactate secretion than MYC non-expressing P493-6 and lactate levels were decreased in both cell lines. This again indicated two independent mechanisms of diclofenac.

**Table 1:** Effects of diclofenac on tumor cells.

	Early effects		Late effects	
	1 h/2 h		24 h/48 h	
	MelIm	U937	MelIm	U937
Intracellular diclofenac	↑*** (0.1)	↑*** (0.1)	n.d.	n.d.
Intracellular lactate	↑*** (0.1)	↑*** (0.1)	↑*** (0.1)	↑*** (0.1)
Extracellular lactate	n.s.	n.s.	↓**/*** (0.1)	↓*** (0.1)
Glucose consumption	n.s.	n.s.	↓***/** (0.1)	↓** (0.1)
Proliferation	n.d.	n.d.	↓*** (0.4)	↓** (0.2) ↓*** (0.4)
MYC protein	↓*(0.8)	n.d.	↓*(0.8)	↓*(0.4)
Cell death	n.d.	n.d.	↑*(0.8)	↑*/** (0.1)
*LDHA* mRNA	n.s.	n.d.	↓** (0.1)	n.d.
*GLUT1* mRNA	n.s.	n.d.	↓** (0.1)	n.d.
*MCT1* mRNA	n.s.	n.d.	↓** (0.1)	n.d.

n.d.: not done; statistical analysis was performed with Student's t-test, n.s.: no significant change; ***p<0.001; **p<0.01; *p<0.05.

Alterations in tumor energy metabolism exert significant effects on tumor growth and metastasis and the use of glycolysis inhibitors alone or in combination with chemotherapeutic drugs has been suggested [63]. Furthermore, the rationale of targeting MYC has been clearly demonstrated by Savino and Soucek [64]; [65], who demonstrated that systemic Myc inhibition by Omomyc, a dominant negative form of Myc, leads to a rapid regression of established tumors [64]. Our findings, that diclofenac inhibits both glucose metabolism and MYC expression, make this well established drug an attractive candidate for inclusion in clinical trials with likely important implications for cancer therapy.

## Supporting Information

Figure S1 **Gemcitabine inhibits proliferation and lactate of the melanoma cell line Mellm.**
(TIF)

Figure S2 **Densitometric analyses of MYC protein expression as a function of diclofenac concentration in Mellm, PC3 and U937 after 24 h.**
(TIF)

Figre S3 **Diclofenac inhibits** ***MCT1*** **,** ***GLUT1*** **and** ***LDHA*** **mRNA expression in Mellm.**
(TIF)

Figure S4 **Accumulation of diclofenac and lactate in U937.**
(TIF)
