# Supplementary figures and images for: New Aspects of an Old Drug – Diclofenac Targets MYC and Glucose Metabolism in Tumor Cells
Source: PLoS One. 2013 Jul 9;8(7):e66987. doi: 10.1371/journal.pone.0066987 (PMC3706586; doi:10.1371/journal.pone.0066987)

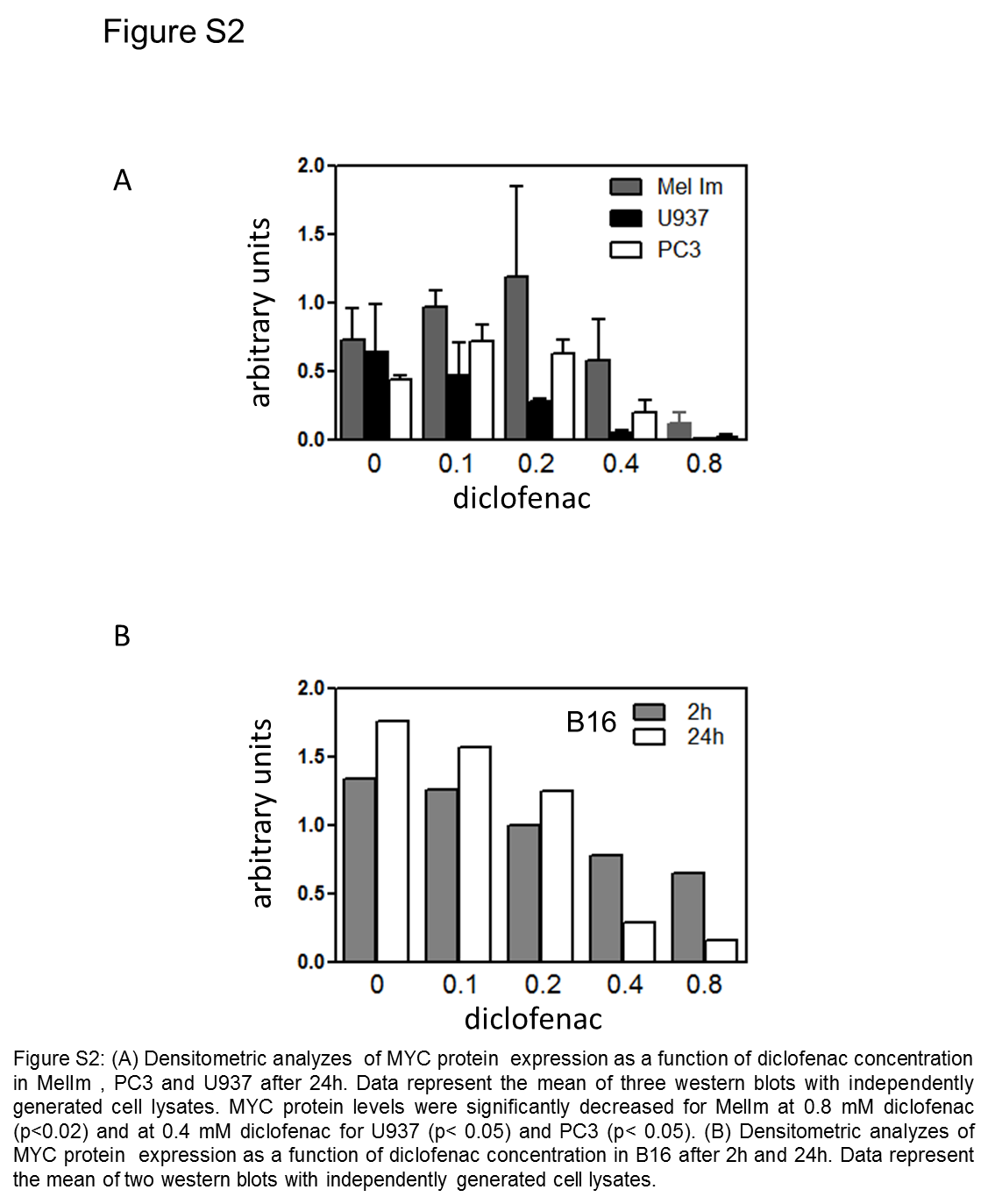

Supplement: Figure S2 — Densitometric analyses of MYC protein expression as a function of diclofenac concentration in Mellm, PC3 and U937 after 24 h. (TIF) [file pone.0066987.s002.tif]
